# Physical Deconditioning as a Cause of Breathlessness among Obese Adolescents with a Diagnosis of Asthma

**DOI:** 10.1371/journal.pone.0061022

**Published:** 2013-04-23

**Authors:** Yun M. Shim, Autumn Burnette, Sean Lucas, Richard C. Herring, Judith Weltman, James T. Patrie, Arthur L. Weltman, Thomas A. Platts-Mills

**Affiliations:** 1 Department of Medicine, University of Virginia, Charlottesville, Virginia, United States of America; 2 Department of Public Health Sciences, University of Virginia, Charlottesville, Virginia, United States of America; 3 Department of Human Services and Department of Medicine, University of Virginia, Charlottesville, Virginia, United States of America; University of Liverpool, United Kingdom

## Abstract

**Background:**

Obese children frequently complain of breathlessness. Asthma and obesity can both contribute to the symptoms during exercise, and this symptom can contribute to a diagnosis of asthma in these children. Despite the high prevalence of obesity few studies have investigated the cardiopulmonary physiology of breathlessness in obese children with a diagnosis of asthma.

**Methods:**

In this case-control study, thirty adolescents between age 12 and 19 were studied with baseline spirometry and a cardiopulmonary exercise test. Ten adolescents were normal controls, ten had obesity without a diagnosis of asthma, and ten had obesity with a history of physician-diagnosed asthma.

**Results:**

Baseline characteristics including complete blood count and spirometry were comparable between obese adolescents with and without a diagnosis of asthma. During exercise, obese asthmatic and obese non-asthmatic adolescents had significantly reduced physical fitness compared to healthy controls as evidenced by decreased peak oxygen uptake after adjusting for actual body weight (21.7±4.5 vs. 21.4±5.4 vs. 35.3±5.8 ml/kg/min, respectively). However, pulmonary capacity at the peak of exercise was comparable among all three groups as evidenced by similar pulmonary reserve.

**Conclusion:**

In this study, breathlessness was primarily due to cardiopulmonary deconditioning in the majority of obese adolescents with or without a diagnosis of asthma.

## Introduction

Over the last fifty years, there has been a steady increase in both asthma and obesity in children [1], [2], [3]. In parallel, there has been an increase in the proportion of overweight children who are diagnosed with asthma [4], [5]. This has led to multiple studies on the mechanisms by which obesity might contribute to asthma [6], [7]. Breathlessness is a cardinal symptom of asthma and is generally thought to be caused by a combination of obstructive lung physiology, inflammation, and/or deconditioning [8], [9]. Even though cardiac failure, anemia, renal failure, and musculoskeletal abnormalities may also present with breathlessness, these chronic diseases are rare among adolescents and asthma becomes the prevailing diagnosis. However, several authors have also recognized the risk of misdiagnosing asthma in obese children [10], [11]. Therefore, the more common problem in adolescent medicine is how to sort out breathlessness related to inflamed airways from other diseases, or as we will argue, obesity related cardiopulmonary deconditioning [12], [13], [14].

There are at least two distinct routes to the development of the combination of obesity and a diagnosis of asthma. First, there are children with inflamed airways causing bronchial reactivity who avoid exercise because it induces bronchospasm and who often require steroid treatment; some of these children will gain weight over time and become obese. Second, there are children who gain weight because of a poor diet and sedentary lifestyle who become progressively short of breath on exercise and receive a diagnosis of asthma because of complaint of breathlessness. Clearly, these two conditions are different, but in practice, they are not distinguished and often are treated in the same way.

Most epidemiological studies on the relationship between obesity and asthma assume that a physician diagnosis of asthma implies the same etiopathogenesis in obese and normal weight children [4], [5], [15], [16]. Studies investigating the specific underlying mechanisms of asthma in obese children or adults, however, have found that obese asthmatics are less allergic and/or less inflamed than normal weight subjects [17], [18], [19], [20]. An important feature of the epidemiological studies is that most of them have used a “physician diagnosis of asthma” or a diagnosis of asthma “based on parental survey” to define which children have asthma [4], [6], [17], [18], [19]. In practice, a diagnosis of asthma is often based on reports of breathlessness and/or wheezing during exercise without objective criteria such as lung function studies. This is because detailed evaluation would require referral to a specialist and because spirometry at rest is often within normal limits [11].

Based on these observations, the objective here was to identify the etiology of exercise limitation and breathlessness by evaluating cardiopulmonary physiology in obese teenagers with or without a history of asthma diagnosis [21], [22], [23]. We used cardiopulmonary exercise testing (CPET) because it: i) offers a testing modality in which the subject is challenged to replicate the subjective symptoms of breathlessness; and ii) provides objective information on the cardio-, pulmonary-, and metabolic- limitation at a time when subjective breathlessness is apparent. The results show that a large proportion of our obese teenage subjects with or without a diagnosis of asthma had no measurable cardiac or pulmonary deficit, and appeared to be limited by deconditioning alone.

## Methods

### Subjects

Prior to enrolment all subjects and their parents signed informed consent documents. The consent procedure and study were approved by the institutional review board of the University of Virginia. Thirty adolescents aged 12 to 19 years were enrolled in three groups: a healthy, non-asthmatic, normal weight control group (controls; n = 10), obese group without history of asthma (OB-CTL; n = 10), and an obese group with a diagnosis of asthma (OB-Asthma; n = 10). Subjects in the latter group had to have been diagnosed with asthma by a physician. Obesity was defined as body mass index (BMI)>95^th^ percentile for age [24]. Anthromorphic measurement was assessed based on published methods [25], [26]. Subjects were asked to discontinue asthma controller medications 24 hours and short acting beta agonists 6 hours prior to physiologic testing. Additional details are provided in an online supplement.

### Baseline assessment and cardiopulmonary stress test (CPET)

Body composition was measured using air displacement plethysmography as described previously [27]. Levels of each subject's physical activity and metabolic rate were calculated by Previous Daily Activity Recall tool (PAR) [28]. Baseline complete blood count (CBC) with differential was obtained before administering CPET. Spirometry (KoKo Spirometry, nSpire Health, Inc.) was obtained before administering CPET and immediately after completing CPET. Exhaled nitric oxide (eNO) and serum total IgE were measured as previously described [29], [30], [31]. Participants completed a modified Balke protocol [32]. At the peak of the exercise each subject's perceived breathlessness was measured by the children's OMNI scale [33]. Data was continuously analyzed to allow interval calculations of spirometry values, including minute ventilation (V_E_), oxygen consumption (VO_2_), carbon dioxide production (VCO_2_), and O_2_ pulse (VO_2_/HR). Maximal voluntary ventilation (MVV) was calculated by multiplying forced expiratory volume in 1 second (FEV_1_) by a factor of 40. Pulmonary reserve (%PR) was calculated as (1-V_E_/MVV)×100 [34]. Additional detail is provided in the online supplement.

### Statistical analysis

Data were analyzed using One-Way ANOVA and Welch's version of the Student's t-test. The degrees of freedom associated with the Welch's t-test were determined via the Satterwaithe approximation method, and a Bonferroni multiple comparison type I error rate corrected rejection rule was implemented in order to restrict the overall probability of falsely rejecting one or more null hypotheses to be no greater than 0.05. The software, MIXED procedure of version 9.1.2 EAS (SAS Institute Inc. Cary, NC) was utilized to conduct statistical analyses.

## Results

### Baseline demographics

The subjects were 14.3±2.4 years old (mean age ± SD) and included 15 males and 15 females. Lean body weight, BMI, fat mass, percent body fat, waist circumference, and sagittal diameter were significantly higher in OB-CTL and OB-Asthma subjects as compared to controls (Table 1). There was no significant difference in baseline spirometry or eosinophils among the three groups (Table 2, Table S1). There was no significant difference in the levels of previous 24 hour-activities quantified by PAR and of estimated metabolic rates among three groups (Table 1).

**Table 1:** Demographic and anthropomorphic characteristics of the study population.

	CTL (n = 10)	OB-CTL (n = 10)	OB-Asthma (n = 10)	P value (CTL vs OB-CTL)	P-value (CTL vs OB-Asthma)	P-value (OB-CTL vs OB-Asthma)
Age (yr)	15.1±2.8*	13.2±1.7	14.7±2.0	0.09† (0.27‡)	0.72 (1.00)	0.09 (0.23)
Gender M/F	5/5	5/5	4/6	1.00 (1.00)	1.00 (1.00)	1.00 (1.00)
Race:						
Caucasian	5	7	3	0.35 (1.00)	0.65 (1.00)	0.07 (0.14)
African American	5	2	7			
Native American	0	1	0			
Weight (kg)	55±14	96±22	115±19	<0.01 (<0.01)	<0.01 (<0.01)	0.05 (0.16)
Height (cm)	161±12	162±5	169±8	0.74 (1.00)	0.11 (0.33)	0.07 (0.20)
Mean BMI	20.7±3.0	36.3±8	40.2±6	<0.01 (<0.01)	<0.01 (<0.01)	0.24 (0.72)
Lean Body Weight (kg)	43.9±10.4	55.4±6.1	62.7±10.4	0.01 (0.03)	0.01 (0.01)	0.08 (0.23)
Fat Mass (kg)	10.6±6.0	42.6±17.8	47.5±11.0	<0.01 (0.01)	<0.01 (<0.01)	0.48 (1.00)
% Body Fat	18.6±7.3	42.0±8.2	45.2±4.6	<0.01 (<0.01)	<0.01 (<0.01)	0.29 (0.87)
Waist Umbilicus (cm)	72.6±8.2	110.3±13.5	130.3±28.1	<0.01 (<0.01)	<0.01 (<0.01)	0.06 (0.19)
Saggittal Diameter (cm)	18.3±1.2	30.2±7.3	35.0±5.1	0.01 (0.01)	<0.01 (<0.01)	0.10 (0.31)
PAR Met Rate (kcal/kg/min)	69.0±24.7	54.0±14.0	60.3±11.6	0.09 (0.12)	0.74 (0.33)	0.12 (0.29)

CTL = healthy normal weight subjects. OB-CTL = obese subjects without a diagnosis of asthma. OB-Asthma = obese subjects with a diagnosis of asthma. PAR Met Rate = Metabolic rate calculated by recall of the previous 24 hour activity in kcal/kg/min. Between-group comparison of means based on the Welch's modified version of the Students t-test. Between-group comparison of frequencies based on the Fisher's exact test. Data are presented as Mean ± SD.

* Mean ± standard deviation,

† Unadjusted p value,

‡ Bonferroni adjusted p value assuming 3 hypothesis tests.

**Table 2:** Spirometry results.

	CTL (n = 10)	OB-CTL (n = 10)	OB-Asthma (n = 10)	P value (CTL vs OB-CTL)	P-value (CTL vs OB-Asthma)	P-value (OB-CTL vs OB-Asthma)
FEV_1_ (Liters)	3.01±0.96*	2.80±0.36	3.17±0.60	0.55† (1.00‡)	0.65 (1.00)	0.12 (0.36)
FEV_1_ % predicted	95±15	95±10	89±17	0.93 (1.00)	0.39 (1.00)	0.37 (1.00)
FVC (Liters)	3.53±1.07	3.38±0.43	4.07±0.93	0.64 (1.00)	0.24 (0.72)	0.05 (0.15)
FVC % predicted	102±15	105±12	103±18	0.90 (1.00)	0.70 (1.00)	0.83 (1.00)
FEV_1_/FVC	0.85±0.07	0.83±0.09	0.79±0.09	0.59 (1.00)	0.10 (0.29)	0.28 (0.83)

CTL = healthy normal weight subjects. OB-CTL = obese subjects without a diagnosis of asthma. OB-Asthma = obese subjects with a diagnosis of asthma. Between-group comparison of means based on the Welch's modified version of the Students t-test. Data are presented as Mean ± SD.

* Mean ± standard deviation,

† Unadjusted p value,

‡ Bonferroni adjusted p value assuming 3 hypothesis tests. FEV_1_ = Forced Expiratory Volume in 1 second. FVC = Forced Vital Capacity.

### Cardiovascular capacities & physical fitness

**Figure 1:**
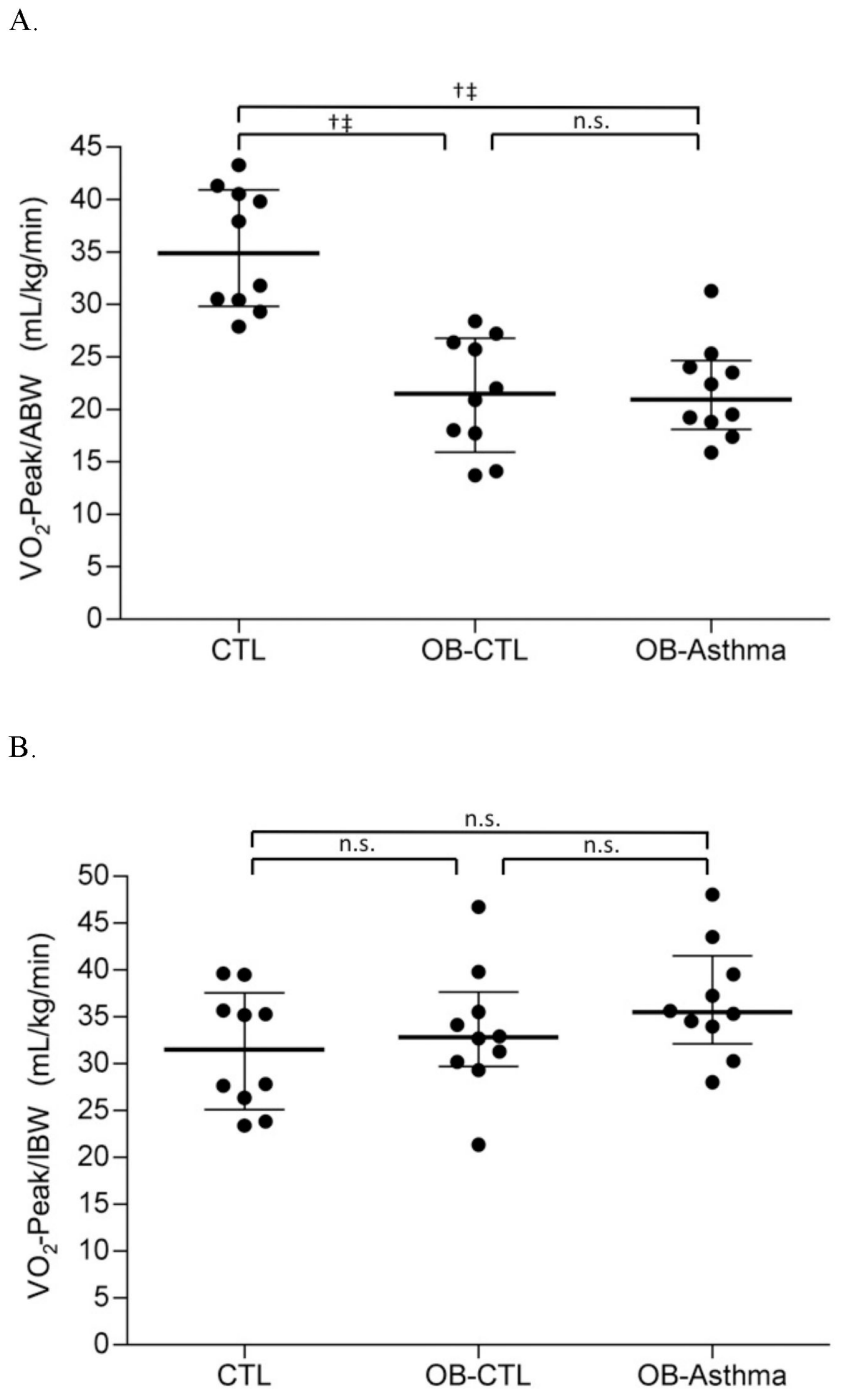
Exercise physiology of O_2_ Uptake. Panel A: Maximum VO_2_ (VO_2_-Peak/ABW) normalized with actual individual weight in kg. Panel B: Maximum VO_2_ (VO_2_-Peak/IBW) normalized with individual ideal body weight in kg. CTL = healthy normal weight subjects. OB-CTL = obese subjects without a diagnosis of asthma. OB-Asthma = obese subjects with a diagnosis of asthma. N = 10 in each group. n.s. indicates p value not significant. † unadjusted p value significant, ‡ Bonferroni adjusted p value assuming 3 hypothesis tests significant.

During exercise testing, the comparable maximum heart rates were achieved among all groups, and each group reached greater than 90% of the percent predicted target heart rate. At the point of volitional fatigue, children's OMNI scores were increased to comparable levels in all three groups (Table 3). Peak Respiratory Exchange Ratios (RER) were also comparable among the three groups (Table 3), and none of the subjects had abnormal electrocardiogram findings during exercise. Overall obese subjects exercised for a significantly shorter duration as compared to controls, but there was no significant difference between the OB-CTL and OB-Asthma subjects (Table 3). Maximum oxygen consumption normalized with actual body weight (VO_2_-Peak/ABW) was significantly lower in the OB-CTL and OB-Asthma subjects as compared to the controls, but no significant difference in the VO_2_-Peak/ABW was detected between the OB-CTL and OB-Asthma subjects (Table 3, Figure 1A). VO_2_-Peak normalized for ideal body weight (VO_2_-Peak/IBW) was not significantly different among the three groups (Table 3, Figure 1B). The O_2_ pulse and the maximum VCO_2_ (VCO_2_-Peak) were not significantly different among all groups in adjusted comparison (Table 3, and online Figure 1).

**Table 3:** Comparison of cardiopulmonary stress test (CPT) results.

	CTL (n = 10)	OB-CTL (n = 10)	OB-Asthma (n = 10)	P value (CTL vs OB-CTL)	P-value (CTL vs OB-Asthma)	P-value (OB-CTL vs OB-Asthma)
Total Exercise Time (sec)	919±215*	515±127	489±149	<0.01† (<0.01‡)	<0.01† (<0.01‡)	0.53 (0.67)
Children's OMNI Scale	7.7±1.9	7.2±2.6	7.7±1.8	0.80 (0.63)	1.00 (1.00)	0.85 (0.62)
Maximum Heart Rate	188.3±14	186.5±11	184±11	0.75† (1.00‡)	0.44 (1.00)	0.60 (1.00)
% Target Heart Rate	91.9±7.0	90.2±5.4	89.6±5.4	0.54 (1.00)	0.42 (1.00)	0.81 (1.00)
Maximum RER	1.06±0.06	1.02±0.07	1.07±0.05	0.14 (0.43)	0.79 (1.00)	0.07 (0.20)
VO_2_-Peak(L/min)	1.93±0.7	2.06±0.4	2.53±0.7	0.64 (1.00)	0.07 (0.19)	0.08 (0.25)
VO_2_-Peak/ABW(mL/min/kg)	35.3±5.8	21.4±5.4	21.7±4.5	**<0.01 (<0.01)**	**<0.01 (<0.01)**	0.89 (1.00)
VO_2_-Peak/IBW(mL/min/kg)	31.4±6.3	33.4±6.7	36.6±5.9	0.51 (1.00)	0.07 (0.22)	0.27 (0.80)
O_2_ pulse(mL/min)	10.3±3.5	11.0±2.1	13.8±3.5	0.56 (1.00)	**0.04 (0.11)**	**0.05 (0.15)**
VCO_2_-Peak(L/min)	2.08±0.79	2.01±0.46	2.60±0.67	0.83 (1.00)	0.13 (0.39)	**0.04 (0.11)**
V_E_(L/min)	63.5±24.2	62.4±14.5	79.0±21.4	0.91 (1.00)	0.15 (0.44)	0.06 (0.18)
Pulmonary Reserve (%)	46.8±12.7	43.2±16.0	37.4±12.4	0.59 (1.00)	0.11 (0.33)	0.37 (1.00)

CTL = healthy normal weight subjects. OB-CTL = obese subjects without a diagnosis of asthma. OB-Asthma = obese subjects with a diagnosis of asthma. OMNI = Children's OMNI scale of perceived exertion. VO_2_-peak = Maximum VO_2_ detected during the CPT. ABW = actual body weight in kg. IBW = ideal body weight in kg [male: 50+(2.3×(height in inches – 60)); female: 45+(2.3×(height in inches – 60))]. VCO_2_ = Maximum VCO_2_ detected during the CPT. RER = Respiratory Exchange Ratio. V_E_ = Maximum minute ventilation detected during the CPT. Between-group comparison of means based on the Welch's modified version of the Students t-test. Data are presented as Mean ± SD.

* Mean ± standard deviation,

† Unadjusted p value,

‡ Bonferroni adjusted p value assuming 3 hypothesis tests.

### Pulmonary capacities

**Figure 2:**
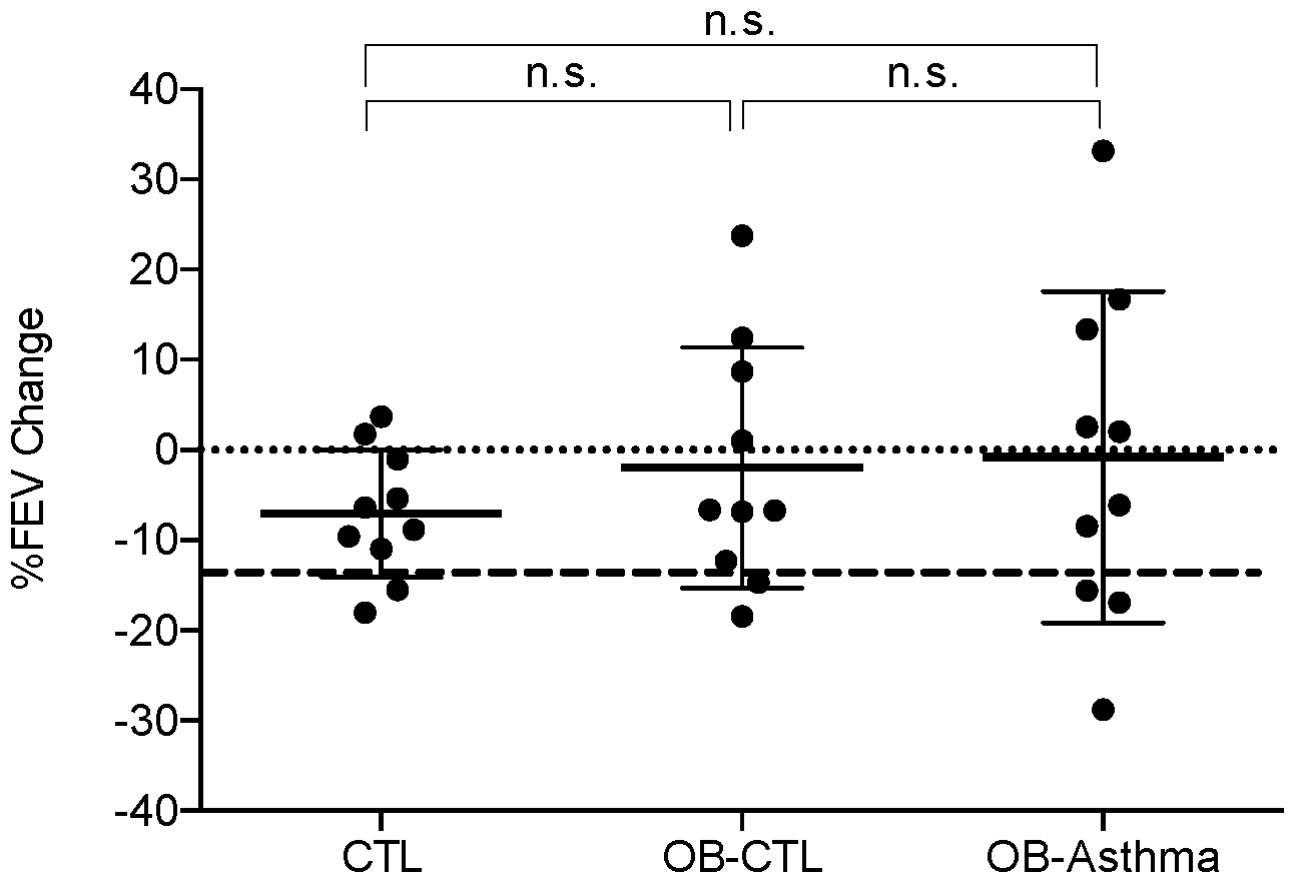
Pulmonary Physiology. Panel A: VE = maximum minute ventilation achieved and measured by continuous spirometry circuit during the exercise. Panel B: Pulmonary Reserve = percentage of unused MVV at the peak of exercise by a formula [%PR = (1−V_E_-Peak/MVV)×100]. CTL = healthy normal weight subjects. OB-CTL = obese subjects without a diagnosis of asthma. OB-Asthma = obese subjects with a diagnosis of asthma. N = 10 in each group. n.s. indicates p value not significant. † unadjusted p value significant, ‡ Bonferroni adjusted p value assuming 3 hypothesis tests significant.

**Figure 3:**
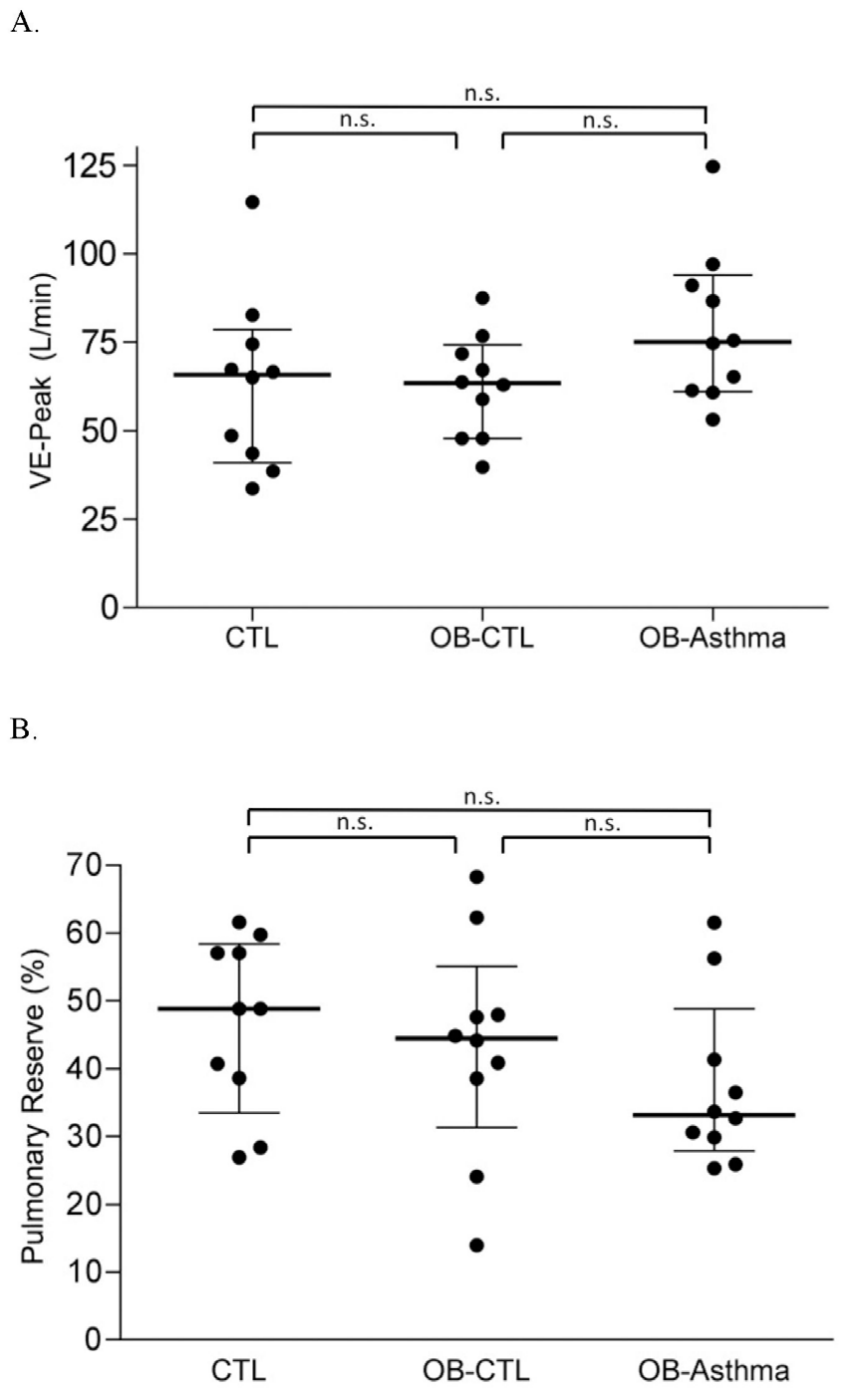
Changes in the FEV1 from ***pre*** **to** ***post*** **CPET.** Percentage changes in FEV1 before and immediately after administering CPET. Thick dotted line = twelve percent reduction in FEV1. N = 10 in each group. CTL = healthy normal weight subjects. OB-CTL = obese subjects without a diagnosis of asthma. OB-Asthma = obese subjects with a diagnosis of asthma. n.s. indicates p value not significant.

The OB-Asthma subjects demonstrated higher levels of V_E_ at the peak of exercise; however, these differences were not statistically different as compared to the controls and OB-CTL subjects (Table 3, Figure 2A). Pulmonary reserve fell below 30% in a comparable number of subjects in each group: two among the controls, two among the OB-CTL, and three among the OB-Asthma (underlined in Table 4). Averages of the %PR among the three groups were not statistically different (Table 3, Figure 2B). The results suggest that only three out of ten OB-Asthma children were restricted by pulmonary capacity, while the same was true for two of the ten in the OB-CTL group. Spirometry was performed before and after exercise, and relative changes in the FEV1 from the values *pre* to *post* exercise were calculated. The changes in FEV1 were not significantly different among three groups (Figure 3). Two among the controls, three among the OB-CTL, and three among the OB-Asthma experienced greater than 12% relative reduction in FEV1 after exercise (data points below the thick dotted line in Figure 3).

**Table 4:** Comparison of V_E_, %PR, eNO, and IgE results.

Group	Study ID	FEV_1_/FVC	%FEV_1_	%FVC	PAR Met Rate	OMNI	V_E_	%PR	eNO	IgE
CTL	1	0.89	106	102	50.5	10	114.6	40.68	8	**741**
	2	0.87	89	94	102.3	5	48.6	61.55	9.3	28.6
	4	0.83	87	96	88.7	7	74.5	28.37	23.5	60.4
	5	0.83	74	81	69.2	6	33.6	48.78	**25.6**	**299**
	6	0.89	82	84	62.1	8	38.5	48.80	12	9.2
	7	0.69	83	110	113.9	9	82.7	26.94	9	4.78
	8	0.88	105	107	40.6	8	43.6	59.78	5.8	26.4
	9	0.94	99	100	52.9	10	65	57.01	18	8.48
	10	0.85	106	117	47.6	5	66.6	38.56	18	**634**
	11	0.85	123	130	61.7	9	67.2	57.03	6.7	27
OB-CTL	14	0.85	84	88	68.9	8	76.8	13.90	22	31.7
	15	0.81	98	112	52.0	10	71.8	38.53	**41**	**445**
	18	0.72	83	106	47.2	7	87.5	24.05	10	101
	19	0.90	100	106	42.7	9	39.7	68.29	14	125
	27	0.88	87	89	86.1	8	63.8	44.81	9	35.9
	29	0.89	101	103	43.2	10	58.9	40.86	11	**624**
	30	0.87	88	91	48.5	8	67.1	47.90	10	19.8
	32	0.75	100	120	43.8	2	63	44.15	11	27.8
	33	0.96	116	109	60.2	4	47.8	62.30	**58**	**182**
	35	0.70	92	121	46.9	6	47.8	47.59	24	103
OB-Asthma	13	0.78	80	97	54.2	10	65.3	36.48	24	**858**
	16	0.78	83	91	48.4	8	97	32.64	20	9.41
	17	0.92	105	109	48.4	6	61.4	56.27	11	81.5
	20	0.84	68	75	63.9	7	60.8	41.31	**55**	**355**
	21	0.83	88	97	76.1	6	75.4	33.63	23	**1370**
	22	0.60	72	111	81.6	10	74.7	25.30	**72**	**182**
	24	0.88	125	130	53.6	9	53.1	61.52	9	13.1
	25	0.72	83	101	50.7	5	86.6	30.61	15	**679**
	26	0.70	104	131	59.7	9	124.6	29.84	**110**	79.3
	31	0.83	82	88	66.0	7	91	25.90	20	**157**

CTL = healthy normal weight subjects. OB-CTL = obese subjects without a diagnosis of asthma. OB-Asthma = obese subjects with a diagnosis of asthma. FEV_1_ = Forced Expiratory Volume 1 second. FVC = Functional Vital Capacity. %FEV_1_ = Percent predicted FEV_1_. %FVC = Percent predicted FVC. PAR Met Rate = Calculated metabolic rate based on recall of previous 24 hour activity (kcal/kg/min). OMNI = Children's OMNI scale of perceived exertion. V_E_ = Maximum minute ventilation detected during the CPT. %PR = Percent pulmonary reserve. eNO = exhaled nitric oxide (ppb). IgE = Immunoglobulin E (IU/mL). Subjects with eNO greater than 25 ppb and or IgE greater than 150 IU/mL are highlighted with underline and bold font. Subjects with %PR less than 30% are highlighted with underline font.

### eNO, total serum IgE, and pulmonary capacity at exercise

In order to assess the relationship between pulmonary capacity at the peak of exercise and markers related to inflammation, values for %PR were reviewed with corresponding eNO and IgE in each patient (Table 4). Based on previously published observations [30], [31], [35], [36], [37], levels of eNO greater than 25 ppb were considered abnormal, and subjects with eNO greater than 25 ppb were marked together with V_E_ and %PR (bold and underlined in Table 4). Levels of eNO were elevated in one control, two OB-CTL, and three OB-Asthma subjects. Two of the three OB-Asthma subjects with elevated levels of eNO had %PR less than 30%. None of the controls or OB-CTL subjects with elevated levels of eNO had %PR less than 30%. Based on usual clinical reference values, levels of total serum IgE greater than 150 IU/mL were considered abnormal, and subjects with IgE greater than 150 IU/mL were marked together with %PR (bold and underlined in Table 4). Three controls, three OB-CTL, and six OB-Asthma subjects demonstrated levels of IgE greater than 150 IU/mL. Three of the six OB-Asthma subjects with IgE greater than 150 IU/mL had %PR less than 30% (25%, 25%, and 29%). None of the controls or OB-CTL subjects with elevated IgE had %PR less than 30%.

## Discussion

Investigation of cardiopulmonary exercise response among obese adolescents found major differences from non-obese controls but no significant differences between those with or without a history of physician-diagnosed asthma. Only a minority of subjects had evidence of respiratory compromise and such individuals were present in all groups. We anticipated that obese children with a diagnosis of asthma would be significantly limited by respiratory abnormalities. Our results suggest that the connection between the activity intolerance and respiratory limitations due to a history of asthma diagnosis was weak in our subjects. Therefore, we believe that breathlessness on exercise should not be taken on its own as evidence of asthma in obese children. The implications as far as management is concerned, however, are more complex than currently appreciated in clinical practice.

Although it is easy to argue that the obese adolescents in our study did not have asthma, we would counter that this is exactly the point. Much of the evidence about an association between asthma and obesity has been based on physician diagnosis or statements such as “asthma was diagnosed using guideline based criteria.” [15], [16], [17], [18] A recent population based study of 17,000 children reported a significant increase of asthma among obese and morbidly obese children. In that study, asthma was defined as following; Asthma status (yes/no) was determined by the parent-completed item, “Did a doctor ever say that (child's name) had asthma?” [4] Where patients have been studied in detail, there exists an increasing body of evidence that obese subjects with a diagnosis of asthma are phenotypically different; they demonstrate less evidence of atopy, lower eNO values, and lower mean eosinophil counts [15], [17], [18], [19].

The results of the present experiments could be taken as evidence to support the view that asthma among obese children represents a different form of the disease [19]. We would question the evidence that this is an inflammatory condition of the airways since breathlessness during exercise in the majority of our subjects was due to cardiopulmonary deconditioning and not to airway changes. There has been a major increase in asthma among all groups of children and also in countries where childhood obesity is much less common than in the United States [2]. In all those countries, lifestyle changes, including the increase in sedentary forms of entertainment, have correlated temporally with the increases in asthma [38], [39]. The question is whether the primary effect of lifestyle on asthma relates to breathing patterns rather than obesity [40], [41]. We believe that breathing patterns, including full expansion of the lungs [41], obesity, and allergen-induced inflammation should all be taken into account when considering the causes of the increase in asthma prevalence [38], [42].

It is important to realize that children with severe asthma and associated inflammation are at risk of becoming obese. In the present study, some children with reduced pulmonary capacity during exercise had elevated eNO and/or IgE. However, a majority of obese children with a diagnosis of asthma were simply deconditioned without any cardiopulmonary abnormalities and had normal levels of eNO and/or IgE. Therefore, we conclude that all children without a contraindication would benefit from an exercise prescription to reverse deconditioning which is likely multi-factorial. It is easy to say that all obese children need an exercise regimen. However, exercise induced bronchospasm can be rapid and severe so that these children will need careful monitoring when they start exercising more vigorously [7], [43], [44]. What is clear from our results is that that those obese children who become breathless on exercise and have normal eNO, are non-atopic, and have normal %PR on exercise, are unlikely to benefit from a treatment regime that includes anti-inflammatory agents and restriction of organized sports [20]. Among the twenty obese teenagers studied here, at least fourteen of the total and seven of those with a diagnosis of asthma would fall into that group.

In this study, we have taken a systematic approach to evaluating the underlying etiology of breathlessness among obese adolescents. This subject is important because of the great number of patients who are diagnosed with asthma, started on inhaled steroids, and cautioned against exertion for fear of asthma exacerbations. Pulmonary function testing and methacholine challenges are available for the objective measurement of asthma. However, these tests can be normal in pediatric asthmatics, particularly if performed at rest. Survey by the PAR suggested that all three groups of subjects had comparable levels of daily activity and of calculated metabolic rates. This led us to believe that a diagnostic study to recreate the subjective breathlessness would be necessary to better identify the etiology of these children's breathlessness. CPET is an unlikely candidate as a routine test. Recruiting overweight adolescents to participate in a four-hour procedure involving body fat measurement and exercise to the point of breathlessness was not easy. However, an exercise evaluation of some kind may be necessary both to help diagnose patients and to guide a starting point for an exercise prescription. As an experimental method, CPET was useful in showing that exercise based measurement of cardiopulmonary reserve can distinguish breathlessness during exercise due to deconditioning from inflamed airways of asthmatics. We paid careful attention to assessing the subjective severity of breathlessness during exercise so that the results of CPET can be temporally correlated with the subjective complaints of breathlessness. These results are not to say that these diagnoses are exclusive, for deconditioning often contributes to breathlessness among patients with asthma caused by inflamed airways.

In conclusion, our results would not change the obvious truth that obese and overweight children need an exercise regime. Some of these children may need pulmonary rehabilitation if difficulty breathing is the perceived reason for not exercising. Based on our results, we propose that cardiopulmonary deconditioning should be considered as an important differential diagnosis for breathlessness among obese adolescents. In addition, we suggest that all studies on the relationship between obesity and asthma that are based on “physician diagnosis,” or even on “guideline-based diagnosis,” should be evaluated knowing that, in as many as 50% of the participants with a diagnosis of asthma, breathlessness on exercise may not reflect a condition of the airways.

## Supporting Information

Figure S1 **Exercise physiology of O_2_ pulse and CO_2_ production.** Panel A: Maximum O_2_-Pulse. Panel B: Maximum VCO_2_ (VCO_2_-Peak). N = 10 in each group. CTL = healthy normal weight subjects. OB-CTL = obese subjects without a diagnosis of asthma. OB-Asthma = obese subjects with a diagnosis of asthma. n.s. indicates p value not significant. † unadjusted p value significant, ‡ Bonferroni adjusted p value assuming 3 hypothesis tests significant.
(TIFF)

Table S1 **Complete blood counts with leukocyte differential percentages and cell counts.** CTL = healthy normal weight subjects. OB-CTL = obese subjects without a diagnosis of asthma. OB-Asthma = obese subjects with a diagnosis of asthma. Between-group comparison of means based on the Welch's modified version of the Students t-test. Data are presented as Mean± SD.
(DOC)

Text S1 **More detailed description of method related to subject recruitment with detailed inclusion and exclusion criteria, protocol for CPET, exhaled NO and IgE measurement, and statistical analysis.**
(DOCX)
