# Supplementary figures and images for: Physical Deconditioning as a Cause of Breathlessness among Obese Adolescents with a Diagnosis of Asthma
Source: PLoS One. 2013 Apr 23;8(4):e61022. doi: 10.1371/journal.pone.0061022 (PMC3634038; doi:10.1371/journal.pone.0061022)

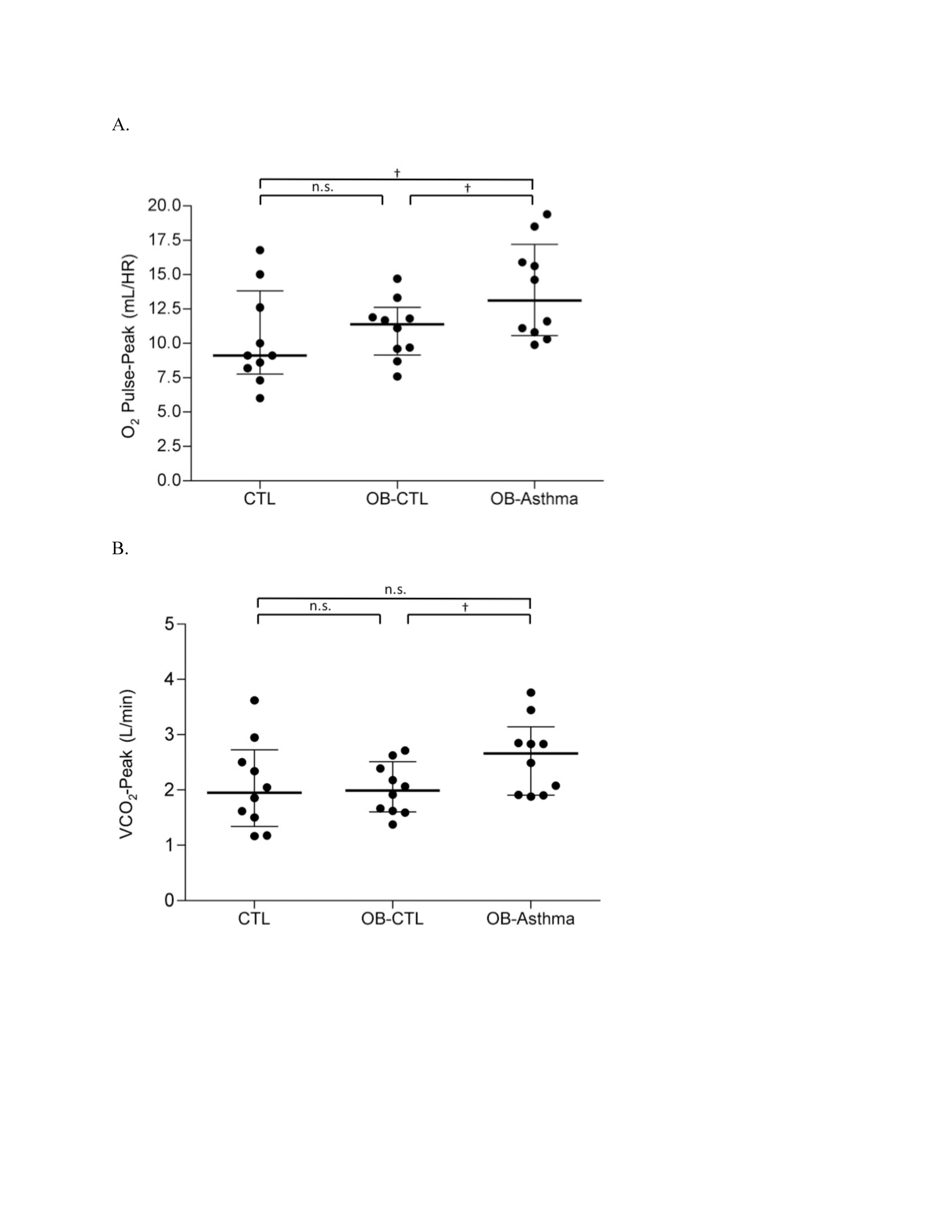

Supplement: Figure S1 — Exercise physiology of O2 pulse and CO2 production. Panel A: Maximum O2-Pulse. Panel B: Maximum VCO2 (VCO2-Peak). N = 10 in each group. CTL = healthy normal weight subjects. OB-CTL = obese subjects without a diagnosis of asthma. OB-Asthma = obese subjects with a diagnosis of asthma. n.s. indicates p value not significant. † unadjusted p value significant, ‡ Bonferroni adjusted p value assuming 3 hypothesis tests significant. (TIFF) [file pone.0061022.s001.tiff]
