# Supplementary material for: Physical Deconditioning as a Cause of Breathlessness among Obese Adolescents with a Diagnosis of Asthma
Source: PLoS One. 2013 Apr 23;8(4):e61022. doi: 10.1371/journal.pone.0061022 (PMC3634038; doi:10.1371/journal.pone.0061022)
Supplement: Table S1 — Complete blood counts with leukocyte differential percentages and cell counts. CTL = healthy normal weight subjects. OB-CTL = obese subjects without a diagnosis of asthma. OB-Asthma = obese subjects with a diagnosis of asthma. Between-group comparison of means based on the Welch's modified version of the Students t-test. Data are presented as Mean± SD. (DOC) [file pone.0061022.s002.doc]

**Table 1.**

|  | CTL (n = 10) | OB-CTL (n = 10) | OB-Asthma (n = 10) | P value (OB-CTL vs. CTL) | P-value (OB-Asthma vs CTL) | P-value (OB-Asthma vs OB-CTL) |
| --- | --- | --- | --- | --- | --- | --- |
| WBC (cells/dL)  Hemoglobin (gm/dL)  Platelets (/dL)  Neutrophil(cells/dL)  Neutrophils(%)  Lymphocyte(cells/dL)  Lymphocytes(%)  Monocyte(cells/dL)  Monocytes(%)  Eosinophil(cells/dL)  Eosinophils(%)  Basophil(cells/dL)  Basophils(%) | 5.6 ± 1.1  14.2 ± 1.4  263 ± 51  2690± 1080  47.7 ± 13.5  2210 ± 790  40.0 ± 12.1  490 ± 120  8.7 ± 2.0  180 ± 100  3.2 ± 1.7  20 ± 10  0.34 ± 0.16 | 7.1 ± 3.0  13.4 ± 1.4  237 ± 31  4490 ± 2590  51.9 ± 11.9  2330 ± 880  31.8 ± 14.1  700 ± 290  9.9 ± 2.5  530 ± 1060  4.5 ± 2.2  38 ± 37  0.44 ± 0.43 | 6.4 ± 2.3  14.3 ± 0.9  303 ± 55  3215 ± 1370  49.7 ± 8.8  2293 ± 889  36.0 ± 6.4  588 ± 176  9.4 ± 1.8  287 ± 148  2.3 ± 1.0  22 ± 13  0.3 ± 0.18 | 0.04† (0.11‡)  0.23 (0.68)  0.65 (1.00)  0.07 (0.20)  0.47 (1.00)  0.77 (1.00)  0.18 (0.54)  0.06 (0.17)  0.84 (1.00)  0.32 (0.95)  0.50 (1.00)  0.14 (0.42)  0.50 (1.00) | 0.33 (0.98)  0.83 (1.00)  0.31 (0.92)  0.36 (1.00)  0.70 (1.00)  0.84 (1.00)  0.37 (1.00)  0.15 (0.44)  0.42 (1.00)  0.07 (0.21)  0.14 (0.42)  0.69 (1.00)  1.00 (1.00) | 0.18 (0.53)  0.12 (0.35)  0.15 (0.46)  0.19 (0.58)  0.64 (1.00)  0.93 (1.00)  0.41 (1.00)  0.33 (0.99)  0.62 (1.00)  0.48 (1.00)  0.80 (1.00)  0.20 (0.59)  0.51 (1.00) |

*mean ± standard deviation, † unadjusted p value, ‡ Bonferroni adjusted p value assuming 3 hypothesis tests.
