# Supplementary material for: Physical Deconditioning as a Cause of Breathlessness among Obese Adolescents with a Diagnosis of Asthma
Source: PLoS One. 2013 Apr 23;8(4):e61022. doi: 10.1371/journal.pone.0061022 (PMC3634038; doi:10.1371/journal.pone.0061022)
Supplement: Text S1 — More detailed description of method related to subject recruitment with detailed inclusion and exclusion criteria, protocol for CPET, exhaled NO and IgE measurement, and statistical analysis. (DOCX) [file pone.0061022.s003.docx]

**Online Text**

**Methods**

*Subjects*

Thirty adolescents aged 12 to 19 years old participated. They were divided into a healthy, non-asthmatic, normal weight control group (CTL, n = 10), a healthy, non-asthmatic, obese group (OB-CTL, n = 10), and an obese group with a diagnosis of asthma based on medical and hospital record (OB-Asthma, n = 10). Subjects responded to advertisements or were recruited from clinics at the University of Virginia Medical Center. Subjects on asthma controller medications were asked to discontinue inhaled corticosteroids, leukotriene antagonists, long acting beta agonists for 24 hours prior to the study or short acting beta agonists for at least 6 hours prior to the study. Subjects were not included if they had had a respiratory infection or had gotten oral corticosteroids within the preceding 6 weeks or if they could not hold their controller medications. Subjects were excluded if pregnant and or anemic by usual clinical reference laboratory tests. Obesity was defined as BMI > 95^th^ percentile for age [[45](#_ENREF_45)]. Asthma was defined as a presence of primary community practitioners’ diagnosis in the medical record. Two subjects were excluded due to low screening hemoglobin defined as <12 g/dL for females and <13 g/dL for males. One subject was excluded due to poor baseline lung function on spirometry defined as predicted forced expiratory volume in 1 second less than 65% which did not improve after bronchodilator treatment. One OB-CTL subject was excluded due to poor effort during exercise which led to unreliable cardiopulmonary exercise test results. After excluding these subjects, subsequent analysis was performed based on results from total 30 subjects. The study was approved by the institutional review board of the University of Virginia Medical Center, and all subjects and their parents signed consent prior to study.

*Baseline assessment*

Body composition was measured using air displacement plethysmography (Bod-Pod, Life Measurement Instruments, Concord, CA) corrected for thoracic gas volume as described previously [[27](#_ENREF_27)]. Standing sagittal abdominal diameter (a measure of visceral obesity that has been correlated with increased risk for coronary heart disease and insulin resistance) was measured with a caliper using the distance from the small of the back to the upper abdomen at the point midway between the top of the pelvis and the bottom of the ribs. Prior to the Cardiopulmonary Exercise Test (CPET), complete blood count (CBC) with differential and baseline spirometry were obtained. Spirometry (KoKo Spirometry, nSpire Health, Inc.) was performed to measure forced vital capacity (FVC), forced expiratory volume in one second (FEV_1_), and FEV_1_ to FVC ratio (FEV_1_/FVC). Percent predicted values of FVC and FEV_1_ (%FVC, %FEV_1_) were derived from the National Health and Nutrition Examination Survey reference. Subjects received two puffs of albuterol by a metered dose inhaler if their FEV_1_/FVC was less than 0.70 and the FEV_1_ was below 80% predicted. Those with normal spirometry and those who demonstrated improvement of the spirometry (i.e. FEV_1_ > 70% predicted) 15 minutes after bronchodilator treatment underwent CPET protocol. Spirometry was measured at 1, 5, 10, and 20 minutes after exercise to evaluate for exercise associated-bronchial hyperresponsiveness.

*Exhaled nitric oxide (eNO) and serum total IgE Measurement*

Exhaled nitric oxide (eNO) and serum total IgE were measured as previously described [[29](#_ENREF_29),[30](#_ENREF_30)]. eNO was collected in all subjects by having the subjects slowly exhale into a NO-impermeable Mylar balloon (Amscan, Harrison, NY). Exhaled gas samples were analyzed for eNO within 1 hour of collection using the Sievers NOA 240 chemiluminescence analyzer (Sievers Instruments, Boulder, CO). As previously described [[29](#_ENREF_29),[30](#_ENREF_30)], serum was separated at room temperature then was processed for total serum IgE using the UniCAP system (Pharmacia Diagnostics, Uppsala, Sweden).

*Cardiopulmonary Exercise Test*

Participants completed a modified Balke protocol. Additional detail on the method for making these measurements is provided in an online data supplement. Maximum VO_2_ (VO_2_-Peak) was chosen as the highest VO_2_ attained during the exercise protocol. The respiratory exchange ratio (RER), heart rate and ratings of perceived exertion, were monitored to ensure that participants attained peak values at the point of volitional exhaustion. Data was continuously analyzed to allow interval calculations of minute ventilation (V_E_), oxygen consumption (VO_2_), carbon dioxide production (VCO_2_), and O_2_ pulse (VO_2_/HR). Maximal voluntary ventilation (MVV) was calculated by multiplying FEV_1_ with a factor of 40. Percent pulmonary reserve (%PR) was calculated as (1-V_E_/MVV) x 100.

*Data Analysis*.

The demographic data, as well as the blood-work data and the spirometry data, and the pulmonary data were analyzed via One-Way ANOVA. In each analysis, the One-Way ANOVA factor was the “study group”, a classification variable with 3 unique levels (CTL, OB-CTL, and OB-Asthma). With regard to hypothesis testing, we elected to use the more robust Welch’s version (20) of the Student’s t-test (i.e. robust with respect to the consequence of violating the equal variance test assumption) as the pivotal quantity in testing the null hypothesis of equal means in the pairwise testing procedure. The degrees of freedom associated with the Welch’s t-test were determined via the Satterwaithe approximation method (21), and a Bonferroni multiple comparison type I error rate corrected rejection rule was implemented in order to restrict the overall probability of falsely rejecting one or more null hypotheses to be no greater than 0.05. The software the MIXED procedure of version 9.1.2 EAS (SAS Institute Inc. Cary, NC) were utilized to conduct the ANOVAs.
